# T-cell/Natural killer-cell neoplasms presenting as leukemia- Case series from single tertiary care center

**Published:** 2016-01-01

**Authors:** Shano Naseem, Maninderbir Kaur, Manupdesh Singh Sachdeva, Jasmina Ahluwalia, Reena Das, Neelam Varma, Subhash Varma

**Affiliations:** 1 Assistant Professor, Department of Hematology, Postgraduate Institute of Medical Education and Research, Chandigarh; 2 Senior Resident, Department of Hematology, Postgraduate Institute of Medical Education and Research, Chandigarh; 3 Associate Professor, Department of Hematology, Postgraduate Institute of Medical Education and Research, Chandigarh; 4 Professor, Department of Hematology, Postgraduate Institute of Medical Education and Research, Chandigarh; 5 Professor and Head, Department of Hematology, Postgraduate Institute of Medical Education and Research, Chandigarh; 6 Professor and Head, Department of Internal Medicine, Postgraduate Institute of Medical Education and Research, Chandigarh

**Keywords:** Morphology, Immunophenotyping, NK-lineage leukemia, T-lineage leukemia

## Abstract

**Background:** Mature T/ NK-cell neoplasms are a rare group of disorders and their presentation as leukemia is even rarer. Most of the previous studies have focused on mature B-cell lineage leukemias and there is a paucity of data on mature T/NK-cell lineage leukemias. We, therefore, planned this study to analyze their spectrum, frequency, morphology and immunophenotypic features.

**Subjects and Methods:** All cases of lymphomas presenting as leukemia over a period of two and a half years were evaluated. Detailed analysis of cases with T/NK-cell lineage was done for their clinical, hematological and immunophenotypic features.

**Results:** A total of 262 cases of mature lymphoid neoplasms presented as leukemia during the study period. Of whom, only 8 (3.1%) cases were of T /NK-cell lineage and the remaining (96.9%) were of B-cell lineage. Of 8 cases, 4 (50%) had T-prolymphocytic leukemia, 2 (25%) had chronic lymphoproliferative disorder- natural killer cell and 1 (12.5%) case of each T-large granular lymphocytic leukemia and hepatosplenic γ/δ T-NHL.

**Conclusion:** T/NK-cell leukemias are rare. Along with clinical and morphological features, pattern of immunophenotypic markers is vital for their diagnosis and subcategorization.

## Introduction

 Leukemias arising from mature T-lymphoid/natural killer (T/NK) cell lineage are relatively uncommon than those arising from B-lineage. The incidence of T/NK-cell neoplasms in a large international study has been reported to be 10-15%; however, variation in incidence in different geographical regions and racial populations is documented.^1^^,^^2^ The mature T-cell neoplasms result from clonal proliferation of post thymic T-cells, which can be either- α/β and γ/δ depending upon type of T-cell receptor (TCR) present on their surface.^3^ α/β subtype is commoner, seen in 95% cases and γ/δ subtype accounts for <5% of mature T-cell. On the basis of the clinical manifestations and origins, mature T-lymphoid neoplasms are classified into three main groups: (i) primary leukemias which arise in the bone marrow (BM) and evolve with a leukemic phase; (ii) leukemia/lymphoma syndrome or the leukemic phase of T-cell lymphomas (T-NHL) which arises in the lymphoid tissues, but a leukemic picture is very common; and (iii) T-NHL, which originates in lymphoid tissues and rarely involves the blood.^4^

Mature NK-cell neoplasms are characterized by persistent proliferation of NK-cells, which are derived from T-cells, but differ immunologically by the absence of TCR gene rearrangements and TCR protein. NK-cells thus express T-cell antigens but are negative for surface CD3; in addition, they also express NK-cell associated antigens-CD16, CD56 and CD57. Mature NK-cell neoplasms presenting as leukemia may have either an indolent course or may be aggressive. They comprise of: (i) chronic lymphoproliferative disorders of NK-cells (CLPD-NK), which have an indolent course and are characterized by persistent (>6 months) increase in peripheral blood (PB) NK-cells (usually ≥ 2x10^9^/L) without a clearly identified cause;^5^ and (ii) aggressive NK-cell leukemias which have systemic neoplastic proliferation of NK-cells and are commonly associated with a leukemic blood picture.^6^ The subtypes of mature T/NK-cell leukemias are outlined in Table 1.

**Table 1:** Subtypes of mature T/NK cell Neoplasms

**1. Mature T-cell neoplasms**
**Primary Leukemias** • T-prolymphocytic leukemias • T-cell large granular lymphocytic leukemia
**Lymphomas with frequent leukemic picture** • Sezary’s syndrome/ mycosis fungoides • Adult T-cell leukemia lymphoma
**Lymphomas with infrequent leukemic picture** • Hepatosplenic γ/δ T-NHL • Subcutaneous panniculitis like T-NHL • Angioimmunoblastic • Extranodal T-cell, nasal type • Enteropathy T-NHL (intestinal) • Anaplastic large-cell lymphoma • Unspecified
**2. Mature NK-cell neoplasms**
**Primary Leukemias** • Chronic lymphoproliferative disorder of NK cells
**Lymphomas with frequent leukemic picture** • Aggressive NK-cell leukemia/lymphoma
**Lymphomas with infrequent leukemic picture** • Extranodal NK-cell lymphoma, nasal type

Most of the previous studies have focused on leukemias arising from mature B-cells and there is a paucity of data on those arising from mature T/NK. We, therefore, planned this study to analyze the spectrum, frequency, morphology and immunophenotypic features of mature T/NK-cell leukemias in our group of patients.

## SUBJECTS AND METHODS

 We analyzed 262 consecutive newly diagnosed cases of mature lymphoid neoplasms presenting as leukemia over a two and a half-year period (January 2011- June 2013). Detailed clinical data was obtained for all the patients. Findings of complete blood counts (performed on LH-750, Beckman Coulter, USA) and morphology of atypical lymphoid cells on PB and BM aspirate smears (May-Grunwald-Giemsa stained) were noted. BM biopsies were evaluated for percentage of cellularity and pattern of infiltration.

Immunophenotyping was performed on PB and/or BM aspirate samples containing adequate number of atypical cells, using combination of fluoroscein isothiocynate (FITC), allophycocyanin (APC), phycoerythrin (PE) and peridinin chlorophyll protein complex (Per-CP) tagged monoclonal antibodies (MoAb). The primary panel included CD19, CD5, CD23, CD20, CD22, CD10, CD38, CD79b, CD43, CD200, FMC-7, kappa, and lambda, CD3, CD4 and CD8. Additional secondary panel was put when the primary panel did yield definite diagnosis and included CD2, CD7, CD1a, TdT, CD16, CD56, CD57, anti-TCR-α/β, anti-TCR-γ/δ, CD25, CD11c and CD103. Staining was done using lyse-wash technique; permeabilization was done for cytoplasmic and nuclear antigens after addition of MoAb for surface antigens. Immunophenotypic analysis was performed on FACS-CANTO-II (BD-Biosciences, San Jose) and a minimum of 10,000 events were analysed. A positive expression was defined based on antigen intensity, when 20% or more lymphoid cells showed fluorescence intensities in excess of the fluorescence intensity associated with the negative control.

The cases were diagnosed and categorized based on the clinical details, morphology and immunophenotypic findings.

## Results

 A total of 262 cases of mature lymphoid neoplasms presenting as leukemia were diagnosed during the study period. 8 (3.1%) out of 262 cases were of T/NK-cell lineage and 254 (96.9%) were of B-cell lineage.

The subtypes seen in these 8 cases of T/NK –cell CLPD included T-prolymphocytic leukemia (PLL) (n=4), CLPD-NK cell (n=2), T-large granular lymphocytic (LGL) leukemia (n=1) and hepatosplenic-γ/δ T-NHL (n=1).

**Clinical Features**

There was a wide range in the age distribution, the youngest patient was a 22-year-old male with CLPD-NK cell and the oldest ones were a 63-year-old female and a male with T-PLL and T-LGL leukemia, respectively. The mean age was 42.5 years. There was a marked male predominance with a male to female ratio of 7:1.

Organomegaly was observed in most of the cases. Hepatomegaly was present in all cases except in one case of T-LGL leukemia. Splenomegaly was also seen in majority of cases except in 1 case of T-PLL and T–LGL leukemia. All cases of T-PLL and hepatosplenic-γ/δ T-NHL presented with lymphadenopathy. It was absent in 1 case of CLPD-NK and T-LGL leukemia. Skin involvement and pleural effusion were seen in 1 case of T-PLL.

**Hematological Features**

Five of the 8 cases had hemoglobin < 10 gm/dL at the time of presentation, hemoglobin levels ranged from 4.5- 13.3 gm/dL (median= 8.9 gm/dL). Thrombocytopenia was seen in all cases except in patient with T-LGL leukemia with a platelet count that ranged from 22- 296 x 10^9^/L (median= 62 x 10^9^/L). Hyperleucocytosis was seen only in T-PLL cases (2 of 4 patients), overall total leucocyte count ranged from 7.5-235.1 x 10^9^/L (median= 13.9 x 10^9^/L).

**Figure 1:**
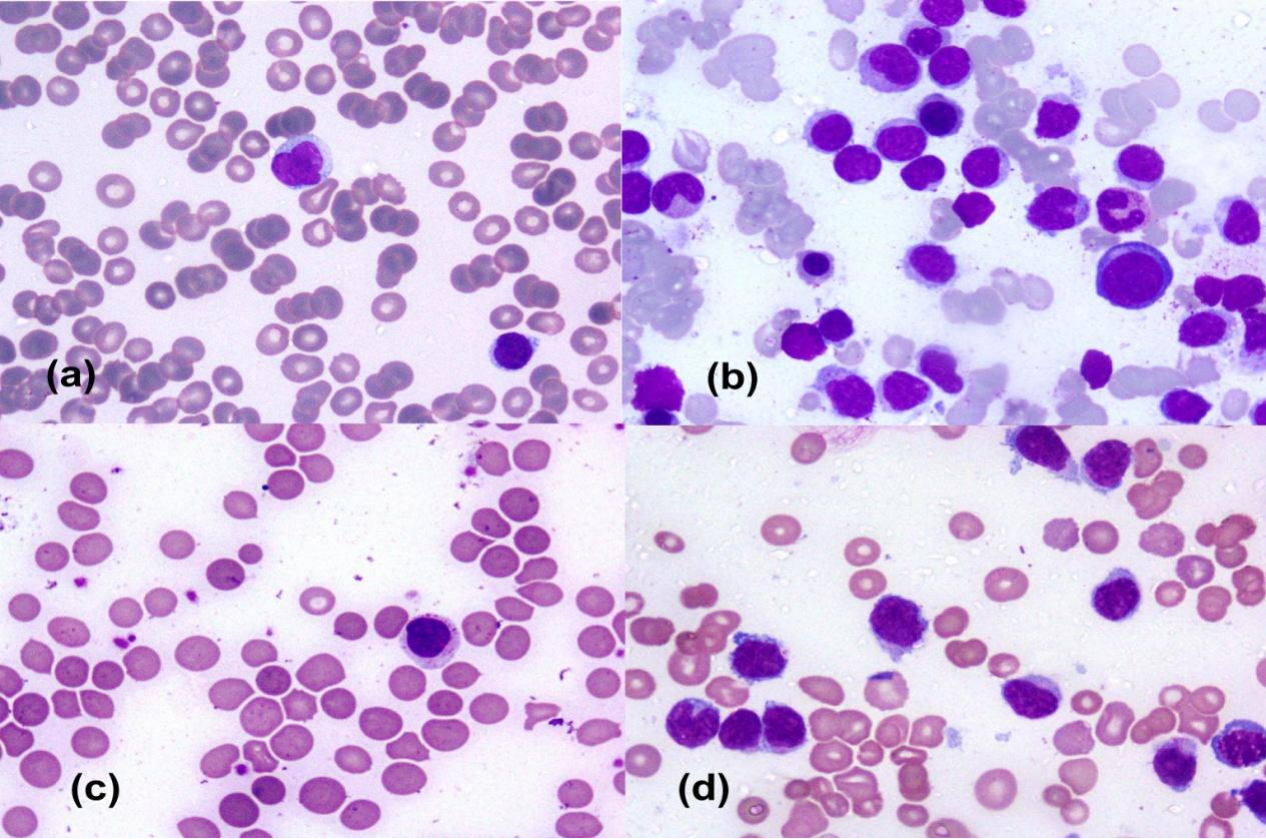
**(a) **T-PLL (cerebriform variant)-Peripheral blood smear showing prolymphocytes with cerebriform nucleus, inconspicuous nucleolus and basophilic cytoplasm with vacuolations; **(b) **CLPD-NK- Bone marrow aspirate smear showing intermediate-sized lymphocytes with condensed nuclear chromatin and moderate amount of cytoplasm with fine granules; **(c) **T-LGL leukemia - Peripheral blood smear showing lymphocytes with moderate cytoplasm and coarse granules; **(d) **Hepatosplenic γ/δ T-NHL –Bone marrow aspirate showing atypical lymphoid cells with opened up chromatin, oval to round nuclei and moderate amount of cytoplasm. (May-Grunwald-Giemsa, 1000X)

 BM was hypercellular in all cases. Three of the 4 cases with T-PLL showed prolymphocytes with dense nuclear chromatin, conspicuous nucleolus and moderate amount of basophilic cytoplasm with blebbing in some of the cells. In one case of T-PLL, the prolymphocytes were smaller in size with cerebriform nucleus having inconspicuous nucleolus and basophilic cytoplasm with vacuolations (cerebriform variant of T-PLL) (Figure 1 a).

**Figure 2:**
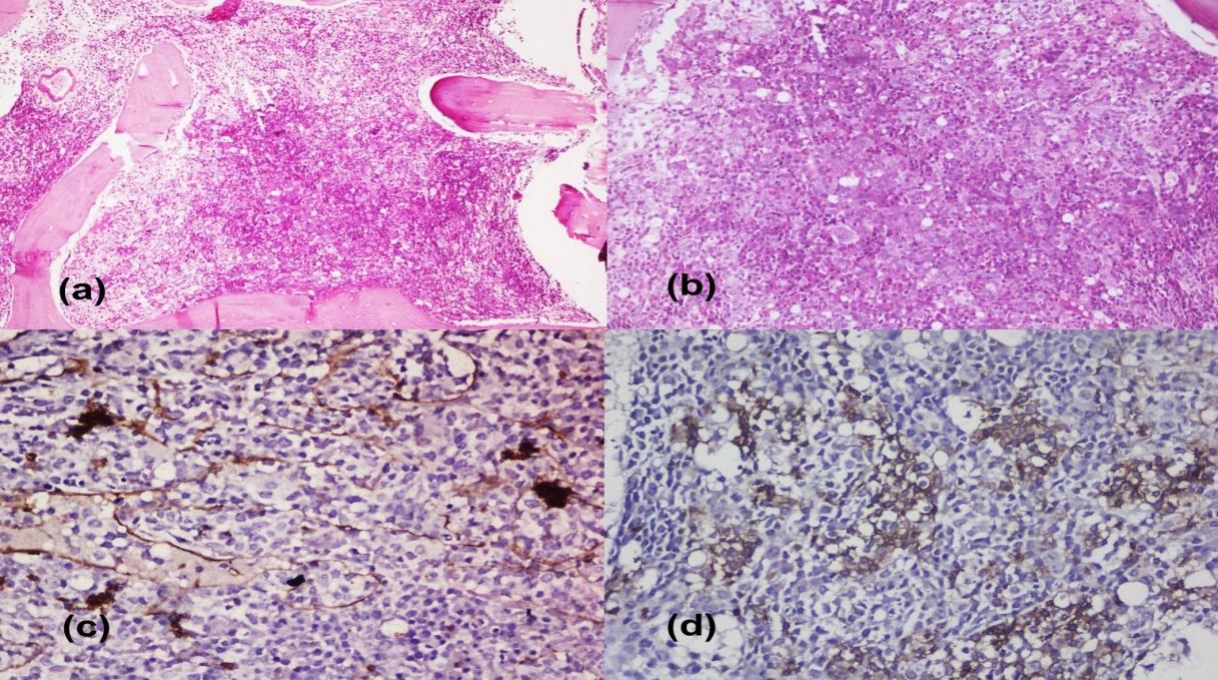
Hepatosplenic γ/δ T-NHL- Bone marrow biopsy showing intrasinusoidal pattern of infiltration [Hematoxylin and Eosin, **(a) **200X, **(b) **400X]. Immunohistochemistry - **(c) **CD34- highlighting the vessels, **(d) **CD3- showing tumor cells within vessels (1000X)

Both cases of CLPD-NK showed intermediate-sized lymphocytes with condensed nuclear chromatin and moderate amount of cytoplasm with fine granules (Figure 1 b). The single case of T-LGL leukemia showed the presence of large atypical lymphocytes with moderate cytoplasm and coarse granules (Figure 1 c). A case of hepatosplenic γ/δ T-NHL showed atypical lymphoid cells with oval to round nuclei and moderate amount of cytoplasm (Figure 1 d). Bone marrow biopsy revealed diffuse and interstitial pattern of infiltration as most common pattern of infiltration. Intrasinusoidal infiltration was seen in 1 case of CLPD-NK cell and single case of hepatosplenic γ/δ T-NHL (Figure 2). 

The details of clinical and hematological features are summarized in Table 2.

**Immunophenotypic Features**

Varied expression pattern of T and NK-cell markers was seen in different subtypes. All the cases of T-PLL were positive for CD3, CD2, CD5, CD7 and TCR-α/β. Two of the 4 cases were CD4+/CD8- and the other 2 were CD4+/CD8+.

Both cases of CLPD-NK cell were uniformly positive for CD7, CD16 and CD56 and negative for CD3, CD4, CD8, CD5, CD57, CD25, TCR- α/β and TCR-γ/δ.

The single case of LGL showed positivity for CD3, CD8, CD5, CD56 and CD16; and the single case of hepatosplenic γ/δ T-NHL showed positivity for CD3, CD7, CD2 and TCR-γ/δ.

**Figure 3:**
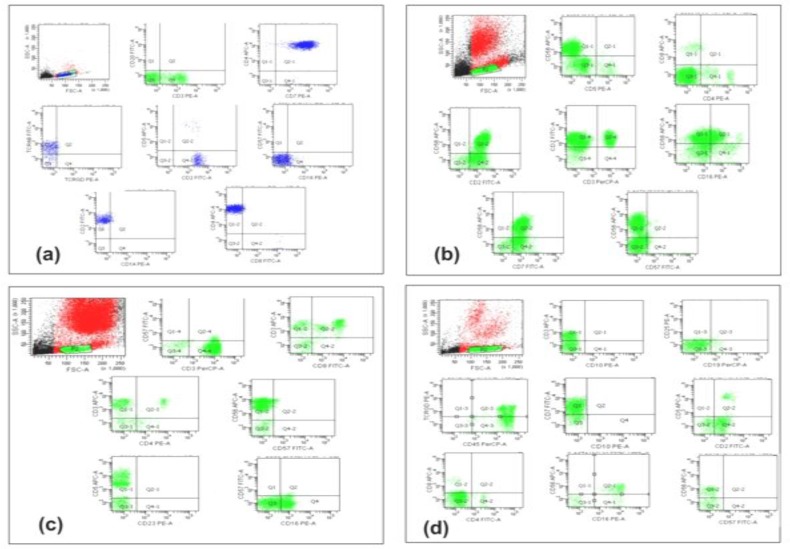
Flow cytometric dot plots of case of **(a)** T-PLL: Positive markers are- CD3, CD4, CD7, CD2 and TCR-α/β; **(b)** CLPD-NK: Positive markers are- CD56, CD16, CD2 and CD7. In addition small proportion normal T-lymphoid cells are also seen; **(c)** LGL: Positive markers are- CD3, CD8, CD5, CD56 and CD16; **(d)** Hepatosplenic γ/δ: Positive markers are CD3, CD7, CD2, CD56 and CD16

The detailed immunophenotypic profile of the cases is outlined in Table 3 and Figure 3 depicts the representative dot plots.

## Discussion

 Amongst the non-Hodgkin’s lymphomas, mature T/NK-cell neoplasms are less common than B-cell, and their presentation as leukemia is further infrequent. There is limited data on the mature T/NK-cell leukemias; however, in this study we analyzed their spectrum, frequency, morphology and immunophenotypic features. 

**Table 2:** Clinical and hematological features of mature T/NK- cell neoplasms presenting as leukemia

	**Case 1**	**Case 2**	**Case 3**	**Case 4**	**Case 5**	**Case 6**	**Case 7**	**Case 8**
**Age/Sex**	31/M	63/F	59/M	35/M	22/M	41/M	63/M	26/M
**Liver**	+	+	+	+	+	+	-	+
**Spleen**	+	+	+	-	+	+	-	+
**Lymph nodes**	+	+	+	+	-	+	-	+
**Skin infiltration**	-	-	+	-	-	-	-	-
**Pleural effusion**	-	+	-	-	-	-	-	-
**Hemoglobin**	11.2	13.3	9.5	11.3	8.3	4.5	6.3	4.8
**TLC x 10** ^9^ **/L**	219.9	235.1	12.6	15.2	12.5	4.6	7.5	37.1
**Platelet count x 10** ^9^ **/L**	76	72	22	39	52	119	296	39
**BM Biopsy- pattern of infiltration**	Diffuse and interstitial	Diffuse	Diffuse	Nodules and interstitial	Interstitial and intrasinusoidal	Interstitial and Nodules	Nodules	Intersitial and intrasinusoidal
**Diagnosis**	T-PLL	T-PLL	T-PLL	T-PLL	CLPD-NK cell	CLPD-NK cell	T-LGL	Hepatosplenic- γ/δ

**Table 3:** Immunophenotypic profile of cases of mature T/NK-cell neoplasms presenting as leukemia

**Markers**	**T-PLL** **N= 4**	**CLPD-NK** **N= 2**	**T-LGL ** **leukemia** **N= 1**	**Hepatosplenic ** **-γ/δ** **N= 1**
**CD3**	100% (4/4)	0% (2/2)	Present	Present
**CD2**	100% (4/4)	50% (1/2)	ND	Present
**CD5**	100% (4/4)	0% (0/2)	Present	Absent
**CD7**	100% (4/4)	100% (2/2)	ND	Present
**CD1a**	0% (4/4)	0% (2/2)	Absent	Absent
**CD16**	50% (1/2)	100% (2/2)	Present	Present
**CD56**	0% (0/2)	100% (2/2)	Absent	Present
**CD57**	0% (0/2)	0% (0/2)	Present	Absent
**CD25**	0% (0/2)	0% (0/2)	Absent	Absent
**CD4+/** **CD8-**	50% (2/4)	0% (0/2)	Absent	Absent
**CD4-/CD8-**	0% (0/4)	100% (2/2)	Absent	Present
**CD4-/CD8+**	0% (0/4)	0% (0/2)	Present	Absent
**CD4+/** **CD8+**	50% (2/4)	0% (0/2)	Absent	Absent
**TCR- α/β**	100% (3/3)	0% (0/2)	ND	Absent
**TCR- γ/δ**	0% (0/3)	0% (0/2)	ND	Present

A total of 262 cases of mature lymphoid neoplasms presenting as leukemia were diagnosed at our center during the study period, of which only 8 (3.1%) cases were of T/NK-cell lineage. A previous study from India also reported a similar incidence of 4% in the cases analyzed by them. All cases in our study were adults. In our study, the median age was 42.5 years with a male predominance and our findings are consistent with previous studies.^7^^-^^9^

WHO in its 2008 classification has outlined 18 subtypes for mature T/NK-cell neoplasms.^10^ However; only few of them have a leukemic presentation, including T-PLL, T-LGL leukemia, Sezary Syndrome/Mycosis Fungoides (SS/MF), adult T-cell leukemia lymphoma (ATLL), CLPD-NK and aggressive NK-cell leukemia. In our study, half of the cases were of T-PLL which constituted 1.5% of total cases of mature lymphoid leukemias, which was similar to findings (2%) reported by Matutes et al. T-PLL is a disease of adults and affects mostly males, with median age of 65 years (range 30–94) at presentation. The principal disease characteristics are organomegaly, skin lesions and a raised lymphocyte count. In addition, skin infiltration and pleural effusion may also be present in some cases. Anemia and thrombocytopenia are common and lymphocyte count of >200 x 10^9^/L is seen in half of the cases.^11^^,^^12^

The median age of our patients was 49 years (range= 31-63 years) with male predominance. Hepatomegaly and lymphadenopathy were seen in all cases, splenomegaly was present in 75% of cases and skin infiltration and pleural effusion were found in 1 case (25%) each. Thrombocytopenia and anemia were present in 100% and 75% of cases, respectively. Absolute lymphocyte count was > 200 x 10^9^/L in 50% of cases.

In our study, 3 cases of T-PLL showed classical prolymphocytes morphology and 1 case showed prolymphocytes with cerebriform nucleus, inconspicuous nucleolus and basophilic cytoplasm with vacuolations. Three morphological variants have been reported in T-PLL. In 75% cases of T-PLL, the lymphocytes have condensed chromatin, regular or irregular nuclear outline, prominent nucleolus and basophilic cytoplasm with blebs (typical T-PLL). In the remaining, the prolymphocytes are smaller in size and the nucleolus is inconspicuous (small-cell variant T-PLL) or has a cerebriform nucleus (cerebriform variant of T-PLL). Immunophenotypically, 50% of our T-PLL cases were CD4+/CD8- and 50% were CD4+/CD8+. Most commonly-T-helper (CD4+ CD8-) phenotype is seen in T-PLL, in two-third of T-PLL cases. Remaining 25% may co-express CD4 and CD8 and cases with CD8+ CD4- are rare. A post-thymic T-cell phenotype with TdT negative, CD2+, CD5+, CD7+ and CD3+ is characteristic of T-PLL.^12^

Although in this study, we did not find any case of ATLL and SS, they need to be considered in the differential diagnosis of T-PLL and T-PLL needs to be morphologically distinguished from B-PLL. ATLL and SS have overlapping morphological features and mature T-cell phenotype. Distinction amongst these is based on a constellation of clinical and laboratory features which include cell morphology, histology and immunophenotype. As compared to T-PLL, lymphadenopathy and skin lesions are uncommon in B-PLL. T-PLL cells differs from B-PLL cells in size (B-prolymphocytes are larger) degree of cytoplasmic basophilia (more in T-prolymphocytes) and nuclear irregularity (irregular in half of the cases of T-PLL). The total leukocyte count is generally below 100 x 10^9^/L and massive splenomegaly is uncommon in ATLL and SS. Other features of differentiation include erythroderma in SS and hypercalcemia and positivity for HTLV-I in ATLL. Morphologically, SS is characterized by cells with cerebriform nuclei and ATLL in the presence of flower cells which have polylobated nuclei. They have some distinctive immunophenotypic features, i.e., SS is negative for CD7 and CD25, ATLL is positive for CD25 and negative for CD7 and T-PLL is positive for CD7 and negative for CD25.

In our study, CLPD-NK cell (the second subtype) was seen in 2 patients. CLPD-NK is characterized by persistent increase in peripheral blood NK cells and was included as a provisional entity in the 2008 WHO classification.^5^ Persistence of NK lymphocytosis for >6 months is important as a transient increase in circulating NK cells may be seen in conditions like autoimmune disorders or viral infections. CLPD-NK occurs predominantly in adults with no sex predilection. The majority of patients are asymptomatic, but some may have systemic symptoms or cytopenias, organomegaly and skin lesions are infrequent.^13^ Both cases of CLPD-NK were seen in males, one was 22 years of age and the other one was 41 years. At the time of presentation, both had anemia, thrombocytopenia and hepatosplenomegaly. Morphologically, both cases had intermediate-sized lymphocytes with condensed nuclear chromatin and moderate amount of granular basophilic cytoplasm. Circulating cells in CLPD-NK are typically intermediate in size with round nuclei with condensed chromatin and moderate amounts of slightly basophilic cytoplasm containing fine or coarse azurophilic granules.^5^ Such large granular lymphocytes may also be seen in T-LGL. The WHO classification recognizes 3 distinct disorders of large granular lymphocytes: T-LGL leukemia, CLPD-NK and aggressive NK-cell leukemia.^14^ T-LGL is characterised by expansion of CD3+ CD8+ cytotoxic T-cells and CLPD-NK by expansion of CD3- cells. Other markers which help to identify NK cell lineage include CD16, CD56, CD57, KIR (CD158), CD94/NKG2A, CD161 and cytotoxic markers for TIA-1, granzyme B, granzyme M.

Bone marrow infiltration may be interstitial or intrasinusoidal and immunohistochemistry may be needed to highlight the infiltration. In one of our cases, the infiltration was interstitial and in the other one both interstitial and intrasinusoidal pattern was seen.

In this study, a 63-year-old male patient was diagnosed with T-LGL leukemia. He had organomegaly, anemia, easy fatigability and fever. Peripheral blood showed 81% LGL which were positive for CD3 and CD8. The trephine biopsy showed a nodular pattern of infiltration. T-LGL like CLPD-NK has an indolent clinical course, one- third of patients are asymptomatic and detected following an incidental finding of cytopenias on a routine blood test. It arises more commonly in patients with autoimmune disorders, particularly rheumatoid arthritis. Most LGL leukemias (80–90%) are CD3 positive with co-expression of CD8, CD16 and CD57, with CD56 and CD28 being negative. TCR-α/β is usually seen and downregulation of CD5 and CD7 may occur.^15^^-^^17^

A single case of hepatosplenic- γ/δ was seen in a 26-year-old male who presented with fatigue, hepatosplenomegaly, lymphadenopathy, anemia and thrombocytopenia. Hepatosplenic T-NHL is an aggressive peripheral T cell lymphoma expressing γ/δ T cell receptors. It has male predominance and occurs predominantly in young adults with median age of 35 years.^18^ Clinically, patients usually have marked splenomegaly and hepatomegaly may be seen in some cases, while lymphadenopathy is uncommon. Patients usually manifest marked thrombocytopenia, often with anemia. BM is involved with an intrasinusoidal pattern;^19^ however, PB involvement is uncommon at presentation, but may occur later. Neoplastic cells are CD3+, TCR- γ/δ+, CD56+/-, CD4-, CD8-/+ and CD5-.^18^

Hepatosplenic- γ/δ T-NHL needs to be distinguished from (i) other NHL subtypes which show intrasinusoidal pattern of BM infiltration including SMZL, intravascular lymphoma, immunophenotyping is useful in differentiation and (ii) other lymphomas with γ/δ T-cells including primary cutaneous γ/δ T-cell lymphoma and extranodal T-cell NHL; however, unlike hepatosplenic γ/δ, they involve extranodal sites- skin and subcutaneous tissue and intestine or nasal regions, thereby making this distinction easy.

## CONCLUSION

 Mature T/NK neoplasms presenting as leukemias constituted 3.1% of all mature lymphoid leukemias in this study. They are seen in adults with male predominance. Most patients had organomegaly, anemia and thrombocytopenia at presentation. Morphology of atypical lymphoid cells showed characteristic features. However, immunophenotyping with limited number of MoAb did not identify the subtype accurately, as there was overlap between the immunophenotypic profiles of different subgroups. Therefore, extended panel of markers including both T-lineage and NK-lineage markers is required in conjunction with clinical details, cell morphology and histology for the diagnosis.
